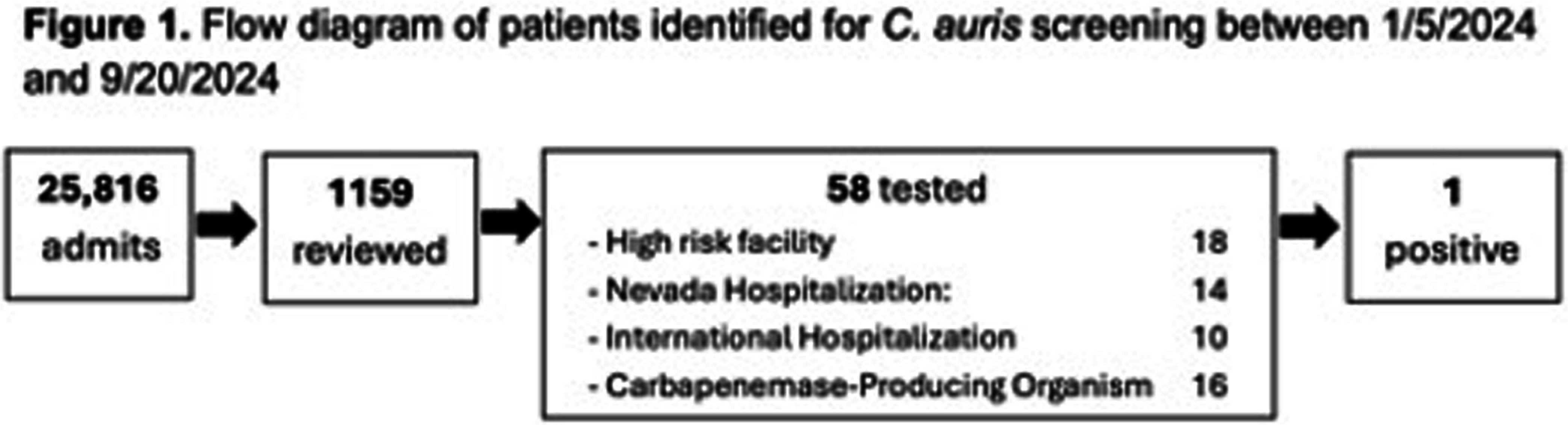# Antimicrobial Resistant Organism Admission Screening Adherence Using a Clinical Information System in a Provincial Healthcare System

**DOI:** 10.1017/ash.2025.418

**Published:** 2025-09-24

**Authors:** Jenine Leal, Zuying Zhang, Logan Armstrong, Janice Pitchko, Bonita Lee, Kristen Versluys, Blanda Chow, Jennifer Ellison, Ted Pfister, Samantha Woolsey, Geraldine St Jean, Stephanie Smith, Elissa Rennert-May

**Affiliations:** 1 Alberta Health Services/University of Calgary; 2 University of Calgary; 3 Alberta Health Services; 4 Alberta Health Services; 5 University of Alberta; 6 Infection Prevention & Control, Alberta Health Services; 7 Infection Prevention & Control, Alberta Health Services; 8 Infection Prevention & Control, Alberta Health Services; 9 University of Alberta

## Abstract

**Background:** Targeted admission screening of high-risk patients for antimicrobial resistant organisms (AROs) is a key component of infection prevention and control. However, adherence with screening is suboptimal, risking a negligible impact on the prevention of ARO transmission. Clinical decision support tools in clinical information systems (CIS) may improve ARO screening adherence. This study evaluated the adherence of ARO admission screening using a tool in the provincial CIS in Alberta, Canada and the relationship between adherence and hospital ARO rates. **Methods:** A population-based, sequential cross-sectional study was completed on all admissions to acute care and acute rehabilitation facilities where ARO admission screening occurs on any unit, and where the CIS was implemented in Alberta between January 1, 2020 and March 31, 2024 (n=100). Mental health facilities/units, continuing care, newborns **Results:** There were 97 (97% of eligible facilities) facilities that implemented the CIS across seven launch periods included. Overall adherence ranged from 43% to 65%. After controlling for bed size and health zone, adherence decreased by the number of months each facility was active on the CIS (aIRR 0.987, 95%CI 0.986-0.987). There was no seasonality in trends. There was a negative relationship between adherence and overall MRSA infection rate (rs = -0.68) and after adjusting for bed size, health zone, and number of months active on the CIS (aIRR 0.99, 95% CI 0.986-0.994). Analysis could not be completed for CPO due to small numbers. **Conclusions:** While increased ARO admission screening adherence was associated with lower overall MRSA infection rates, the IRR was close to one and may not be clinically significant. With adherence decreasing over time, further work is needed to understand barriers to ARO admission screening and implement strategies to support healthcare providers in completing appropriate surveillance for AROs.

**Figure:**